# Differences in agricultural activities related to incidence of scrub typhus between Korea and Japan

**DOI:** 10.4178/epih.e2017051

**Published:** 2017-11-09

**Authors:** Chang-Jin Ma, Gyung-Jae Oh, Gong-Unn Kang, Jeong Mi Lee, Da-Un Lee, Hae-Sung Nam, So Yeon Ryu, Young-Hoon Lee

**Affiliations:** 1 Department of Environmental Science, Fukuoka Women’s University, Fukuoka, Japan; 2 Department of Preventive Medicine and Public Health, Wonkwang University School of Medicine, Iksan, Korea; 3 Department of Medical Administration, Wonkwang Health Science University, Iksan, Korea; 4 Graduate School of Public Health, Wonkwang University, Iksan, Korea; 5 Department of Korean History Education, Wonkwang University College of Education, Iksan, Korea; 6 Department of Preventive Medicine and Public Health, Chungnam National University School of Medicine, Daejeon, Korea; 7 Department of Preventive Medicine, Chosun University Medical School, Gwangju, Korea

**Keywords:** Scrub typhus, Knowledge, Behavior, Agriculture, Korea, Japan

## Abstract

**OBJECTIVES:**

The purpose of this study was to establish a basis for improving or strengthening the preventive strategy against scrub typhus in Korea by comparing and analyzing the difference of prevention behaviors contributing to the occurrence of scrub typhus in Japan and Korea.

**METHODS:**

The survey was carried out in Jeollabuk-do, which is a high risk and high incidence area, and Fukuoka Prefecture, which is a high risk and low incidence area. The study included 406 Korean farmers and 216 Japanese farmers. Data were collected through face-to-face surveys by interviewers who had completed standardized education.

**RESULTS:**

Korean farmers have a higher percentage of agricultural working posture that involved contact with weeds than Japanese farmers (p<0.05). The frequency and proportion of weeding were lower in Korean farmers than in Japanese farmers (p<0.05). The level of knowledge about scrub typhus was significantly higher among Korean farmers than among Japanese farmers (p<0.05). Mostly, the behavior of agriculture work was more appropriate for Japanese farmers than for Korean farmers (p<0.05). The total average level of agricultural work was lower in Korea than in Japan, lower in men than women, and lower in part-time farmers than full-time farmers (p<0.05).

**CONCLUSIONS:**

This study suggests that it is reasonable to develop and provide a program that can improve the level of preventive behavior taking into consideration the characteristics of the subject in order to reduce the incidence of diseases in high-risk areas for scrub typhus.

## INTRODUCTION

Korea and Japan are within the high-risk area for scrub typhus. The southern regions of the two countries, in particular, do not exhibit a significant difference in the climate, ecological environment, and demographic characteristics of affected patients [1-5]. Scrub typhus in the southern parts of Korea and Japan was more prevalent in farmers or people involved in agricultural work, females, and individuals aged 60 years and above. Temporally, it occurred mainly in the autumn [3,6]. Considering that autumn is the season when agricultural work related to harvest mostly occurs and that scrub typhus patients are commonly related to agricultural work by nature, the occurrence of scrub typhus in the southern regions of Korea and Japan can be considered closely related to agricultural tasks performed during the autumn.

The epidemiological characteristics of scrub typhus in the southern regions of Korea and Japan are similar, but there is a big difference in the incidence rate between the two countries. The cumulative number of cases and incidence rate per 100,000 population in the past 10 years (between 2007 and 2016) were quite low at 4,185 and 0.32, respectively, in Japan, whereas in Korea, they were very high at 75,658 and 14.92, respectively. The cumulative number of scrub typhus cases was 18 times higher in Korea than in Japan, and the incidence rate per 100,000 population was 47 times higher in Korea than in Japan, contributing to a greater burden of disease due to scrub typhus [7-9]. The reason for the increased incidence of scrub typhus in Korea after 2000 is reportedly related to the increase in pathogens and vectors due to global warming, as well as the increase in exposure behaviors [10]. With regards to agriculture, the increase of eco-friendly farming methods, the increase in cultivation rate, and the aging agricultural population can be considered [11-14].

Scrub typhus is an infectious disease that is transmitted by mites. In order to prevent and control scrub typhus, avoidance of exposure to the vector should be considered first and foremost [15]. Considering the similarities in the environmental and climatic conditions contributing to the incidence of scrub typhus in the southern regions of Korea and Japan, as well as the fact that the main vector (*Leptotrombidium scutellare*) is similar between the two countries [1-6], the difference in the incidence of scrub typhus between both countries must arise from characteristics related to human-vector contact. However, studies conducted so far have yet to produce solid evidence regarding the prevention and control of scrub typhus through contact interference with mites in high-risk groups [5,16], and the implementation of active management policies has been limited.

Therefore, establishing the difference in work behavior or environmental management related to scrub typhus incidence between the two countries and devising a solution to interfere with mite contact in high-risk agricultural workers would decrease the incidence of scrub typhus in Korea [17]. This study aimed to clarify the difference in agricultural work behavior related to the interference and avoidance of contact with scrub typhus vector between Korea and Japan, thereby suggesting an effective preventative strategy for the reduction in scrub typhus incidence in Korea.

## MATERIALS AND METHODS

### Study subjects

In order to increase the comparability between the two target areas, high-incidence areas in Korea and a low-incidence area in Japan with a similar risk of occurrence in terms of geographic, climatic, and agricultural characteristics were selected. According to this standard, Jeollabuk-do in Korea, which is a high-risk, high-incidence area, and Fukuoka Prefecture in Japan, which is a high-risk, low-incidence area, were selected.

Jeollabuk-do and Fukuoka have a similar distribution of plains and rolling hills. The average annual temperature, precipitation, and farmland composition of Fukuoka were similar to those of Jeollabuk-do [18-20]. Fukuoka is considered a high-risk area for scrub typhus in terms of geological and climatic characteristics, but the 10-year incidence of scrub typhus in Fukuoka was only 25 cases, which was the lowest of all prefectures within Kyushu [7]. Fukuoka is the most similar of all prefectures in Kyushu in terms of geological and climatic characteristics to Jeollabuk-do, but the incidence status of scrub typhus in the two regions showed a stark contrast, making Fukuoka an ideal target for the comparison of factors related to scrub typhus incidence [7,9].

**Figure 1.**
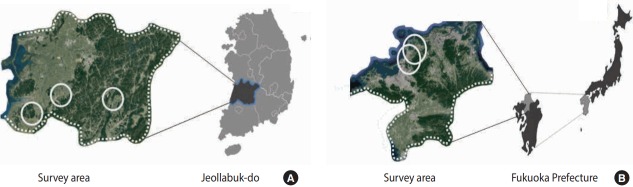
The surveyed areas consisted of 7 villages (3 regions-white open circles) in Jeollabuk-do in Korea (A) and 5 villages (2 regions-white open circles) in Fukuoka Prefecture in Japan (B). These areas were similar regarding agricultural characteristics, agricultural work environment, and topography.

Villages were set as the target unit for survey considering the agricultural characteristics and environment, in order to maintain homogeneity in the characteristics and environment of agricultural work as well as geographical environment and behavior. Villages were non-randomly selected using judgment sampling, after analyzing the geological characteristics, agricultural land and crop types, and the ability to engage and participate in the survey via expert discussion within healthcare facilities and agricultural organizations, as well as prior field survey. Seven villages across 3 areas in Korea and 5 villages across 2 areas in Japan were selected for analysis (Figure 1).

Subjects were limited to those working in the agricultural field regardless of the extent of work. In all, 406 Korean subjects and 216 Japanese subjects were selected for analysis.

### Study design

Data were collected via face-to-face surveys by interviewers who completed standardization education. Common survey tools and guidelines were developed and used for the standardization of the surveys, and the interviewers were educated through a common educational program. The surveys were conducted for a month between late November 2014, and mid-December 2014, considering the climate, the spike of scrub typhus incidence in October and November, and the end of harvest time around December in Jeollabuk-do and Kyushu [3,6,21].

The criteria for the survey period were confirmed through a pilot survey. For agricultural characteristics, ‘past 3 months’ was applied, considering that September to November is the harvest period with the highest agricultural workload, and that *L. scutellare* larvae, the main vector for scrub typhus, are particularly active during this period. ‘Past 6 months’ was applied to parameters related to weeding, considering the period during which most of the weeding occurs. Standardized survey tools and guidelines were developed first in the Korean language, and then translated into Japanese by a Korean translator who majored in Japanese. Then the translated Japanese documents were again translated by a Japanese translator who majored in Korean. The survey categories included population sociological characteristics, agricultural work experience, the average length of daily agricultural work, the level of knowledge on scrub typhus, agricultural work behavior, and weeding characteristics. The level of knowledge on scrub typhus was measured by the percentage of correct answers to each question, and the response ‘I don’t know’ was considered incorrect. Agricultural work behavior related to scrub typhus was analyzed using a 5-point Likert scale.

### Data analysis

All statistical analyses were performed using SPSS version 21.0 (IBM Corp., Armonk, NY, USA). The between-country difference in distribution of patient characteristics, mid-work breaks, the proportion of weeding, and the level of knowledge was evaluated using the chi-square test, and the between-country difference in agricultural work experience, work posture, the frequency of weeding, the total knowledge score, and agricultural work behavior were analyzed using the t-test. The between-country difference in agricultural work behavior adjusted for the general subject characteristics was analyzed using two-way analysis of variance. The p-values of less than 0.05 were considered statistically significant.

### Ethics statement

The present study protocol was reviewed and approved by the Wonkwang University institutional review board (IRB no. WKIRB-201410-SB-059).

## RESULTS

### General characteristics of the study subjects

A total of 406 Koreans and 216 Japanese subjects were included in the analysis. In Korea, subjects in their 70s were the largest in proportion (202 subjects, 49.8%), whereas, in Japan, subjects in their 60s were the largest in proportion (108 subjects, 50.0%), showing a statistically significant difference in the age distribution between the two countries (p< 0.001). Sex distribution also showed statistically significant difference, with 63.5% of Korean subjects being female, while 66.2% of Japanese subjects were male (p<0.001). The proportion of professional farmers was 98.8% in Korea and 79.6% in Japan, again showing a significant difference between the two countries (p< 0.001) (Table 1).

### Characteristics of agricultural work and weeding

The average length of experience in agricultural work was 43.2 years in Korea and 30.5 years in Japan, and the average length of daily agricultural work was significantly longer at 8.0 hours in Korea and 5.2 hours in Japan (p< 0.001). Agricultural work posture that potentiates contact with grass, such as bending forward, kneeling and crouch, was more common in Korea than in Japan (p<0.001) (Table 2).

There was a between-country difference in the distribution of the rest area during agricultural work (p< 0.001) (Table 2). In Korea, 73.3% of the rest area was found around the farmland, and home comprised 23.5%. In Japan, 36.7% of the rest area was around the farmland, 35.6% was home, and 24.9% were interiors of machinery or cars.

There was no difference in the proportion of weeding between Korea and Japan at 86-89%, but weeding around the living area was lower in Korea (71.7%) than in Japan (93.1%) (p< 0.001). The frequency of weeding per 2 months was lower around the farmlands in Korea (3.1 times) than in Japan (3.8 times), as well as around the living area in Korea (1.8 times) than in Japan (2.6 times) (p< 0.05) (Table 2).

### Level of knowledge on scrub typhus

The general level of knowledge on scrub typhus (time of incidence, transmission pathway, risk factors, clinical symptoms, postexposure management and other characteristics, major prevention behaviors, and the use of mite repellant) was relatively higher in Korea than in Japan (p< 0.001). Korean subjects showed high scores at 60-90 in most categories such as the time of incidence, vector, symptoms, prognosis, the use of protective agricultural gear, and post-exposure management. However, they showed low scores below 60 in the categories of household agricultural work, the length of the dormant period, recurrence rate, human-to-human transmission and the use of mite repellant. Japanese subjects scored below 30 in most categories but scored higher at 30-50 in the categories of the use of agricultural safety gear and post-exposure management. In particular, the level of knowledge on personal hygiene, including the use of agricultural safety gear, washing of agricultural work gear, and bathing, tended to be higher than the national average in both Korea and Japan (Table 3).

### Agricultural work behavior

The general level of agricultural work tended to be lower in Korea than in Japan (p< 0.001). In particular, the use of hat, neck towel, long-sleeves or sleeve covers, and boots, the avoidance of laying down clothes on grass and sitting or lying on grass, the washing of clothes after agricultural work, and showering or bathing after agricultural work were lower in Korea than in Japan (p<0.001). The use of neck towel was low in implementation level compared to other categories in both countries. On the other hand, the use of gloves, long pants, and socks did not show a significant difference between the two countries (Table 4).

**Figure 2.**
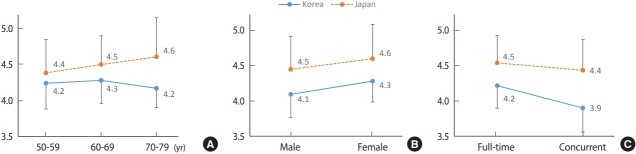
Differences in total average level of behavior of agriculture work according to general characteristics and country. Data were expressed as the means and standard deviation. Statistical analysis were performed using two-way analysis of variance to determine the main effects and interaction between variables. (A) F_country_=47.044, p<0.001; F_ages_=1.503, p=0.22; F_country×ages_=4.978, p=0.007. (B) F_country_=77.280, p<0.001; F_sex_=19.568, p<0.001; F_country×sex_=0.224, p=0.64. (C) F_country_=18.541, p<0.001; F_agricultural type_=4.659, p<0.05; F_country×agricultural type_=2.953, p=0.09.

The behavior of agricultural work was lower in general across all ages in Korea compared to Japan (p< 0.001). Among Korean subjects, the score was highest in those in their 60s and lower in those in their 50s and 70s; however, among Japanese subjects, the score increased with age (p< 0.001). The behavior of agricultural work was lower in general across genders in Korea than in Japan (p< 0.001) and higher among females than males in both countries (p< 0.001). On the other hand, behavior of agricultural work was lower in Korea regardless of whether the subjects were full-time agricultural workers or not. In Korea, part-time agricultural workers scored lower than full-time workers (p< 0.05). However, in Japan, there was no difference in scores between part-time and full-time agricultural workers (Figure 2).

## DISCUSSION

In general, older agricultural workers tend to be more susceptible to infection due to lower immune capability and rest more often near the farmland due to lower physical stamina. Female agricultural workers tend to work manually in the fields where mites proliferate, increasing the probability of contact with mites [13,22]. Fieldwork tends to involve squatting or bending forward, which increases contact with mites that live in the bushes, crops, and soil and heighten the risk of bites [22].

Subjects tended to be older and higher in the proportion of females and professional agricultural workers in Korea than in Japan. The average length of daily agricultural work tended to be longer, and the proportion of bending forward or squatting tended to be higher in Korea than in Japan. Therefore, based on the population characteristics and the characteristics of agricultural work, Korean subjects tend to be more susceptible to mite contact and have higher risk of scrub typhus incidence.

Agricultural land and the surrounding areas as well as the areas surrounding agricultural villages are main areas in which human-to-mite contact occurs because agricultural workers inhabit these areas where mites proliferate due to the abundance of crops or bushes [22,23]. Mid-work rest in Korea occurs mostly around farmlands, such as the banks of rice paddies and fields, which is different from Japan, where it occurs mostly in cars and homes. The banks of rice paddies and fields are major areas populated by mites, the main vector of scrub typhus. Hence, resting in such places in between agricultural work increases human-to-mite contact [24].

Mites have a low infection rate to scrub typhus at 1-2% and tend to dwell in select areas called mite islands [16,25,26]. Due to such characteristics, it is difficult to identify mites infected by scrub typhus and to recognize the areas inhabited by mites. Therefore, eliminating mites for the prevention of scrub typhus would not be so efficient. Field use of pesticides must be carefully approached, considering the financial burden and environmental contamination that can occur due to pesticide use.

For these reasons, minimizing the opportunity for human-to-mite contact by eliminating mite habitat within the living areas, rather than pest control, may be more effective for the prevention of scrub typhus [27]. Studies show that weeding around the living quarters tend to be inadequate in Korea, where the incidence rate of scrub typhus is high, compared to Japan, where the incidence rate is low. Therefore, the elimination of mite habitats around the living areas through weeding can be considered a preventive measure against scrub typhus.

Proper adherence to preventive strategies such as avoidance of mite contact and removal of infiltrated mites is known to be the most effective preventive measures for infectious diseases transmitted by mites [15,28]. In this study, the level of agricultural work tended to be lower in Korea than in Japan. In particular, the use of agricultural gear that minimized skin exposure, work behavior that minimizes contact with bushes to prevent mite contact, removal of mites in clothes or skin through personal hygiene such as washing of work clothes or bathing tended to be lower in Korea. Therefore, Korean agricultural workers tend to be at a higher risk for mite bites due to greater opportunity for mite contact or mite infiltration. The relatively low levels of agricultural behavior in Korea, where the incidence of scrub typhus is high, show that the behavior in agricultural work can explain the high incidence of scrub typhus.

Health-related behaviors are known to be habituated over a long period under the influence of social and cultural characteristics [29]. Subjects from Fukuoka showed lower rate of skin exposure during agricultural work despite the average annual temperature that is around 4°C higher than Jeollabuk-do (in 2012) [20]. In subjects older than 70 years, female subjects, and full-time agricultural workers, who are at a higher risk for mite exposure, the level of agricultural work behavior tended to be higher in Japan than in Korea. These results show that the level of work behavior can differ from scrub typhus risk factors and incidence. Therefore, agricultural work behavior can be established under the influence of social and cultural characteristics to a degree, in addition to the awareness of scrub typhus or climatic factors. Appropriate levels of prevention can be expected from the improvement of agricultural work behavior for the prevention of scrub typhus, only with active reflection of socio-cultural characteristics within the high-risk areas and high-risk subject groups.

The level of agricultural work behavior tended to increase with age, whereas it was lower in Korean subjects in their 50s and 70s compared to Korean subjects in their 60s. Therefore, more focused management of the relatively younger population as well as elderly population should be implemented in Korea, and the identification and removal of impeding factors that prevent them from adopting appropriate agricultural work behavior are crucial.

In both Korea and Japan, the level of agricultural work behavior in males or part-time agricultural workers tended to be lower than females or full-time workers. In high-risk areas such as Korea, all agricultural workers, regardless of the nature of their work and their demographic characteristics, are at high risk for exposure to scrub typhus risk factors. Intervention measures for the prevention of scrub typhus in high-risk areas must include male or parttime agricultural workers, since low levels of agricultural behaviors in these populations can increase the incidence of scrub typhus. The results show that Korean subjects have higher levels of knowledge on scrub typhus but lower levels of agricultural work behavior compared to Japanese subjects. Since the incidence rate of scrub typhus is maintained at a higher level in Korea than in Japan, inhabitants of high-risk areas in Korea tend to have a higher level of disease awareness as well as a greater amount of exposure to disease-related education and campaign. This may be the reason behind the higher level of knowledge on scrub typhus shown in this study. On the other hand, these results show that the high level of knowledge on scrub typhus is not appropriately connected to the implementation of preventive activities against scrub typhus. Education is reported to be the most effective intervention to increase the rate of adherence to preventive measures if the level of knowledge on mite-mediated diseases is high [17,30,31]. Therefore, in order to minimize the incidence of scrub typhus in high-risk areas such as Korea, education programs that can increase the adherence rate to preventive measures, in addition to increasing the knowledge on scrub typhus, are crucial.

This study focuses on behavioral characteristics, among the many factors that can influence the incidence of scrub typhus and does not suggest a preventive strategy that encompasses the distribution and habitation of vectors and terminal carriers or climate. This study used a judgment sampling approach, rather than a probability sampling method, to randomly select areas and subjects suitable for inclusion in the analysis; therefore, the representativeness of the sample might be limited. In addition, when contemplating the results of this study, one must consider that age, sex, and degree of participation in agricultural work may have influenced the low level of agricultural behavior in Korea compared to that in Japan, since the demographic characteristics of the subjects differ between the two countries.

Despite these limitations, the results of this study are significant, since they highlight the difference in the environmental management and the behavioral pattern related to mite contact in two countries with similar risk but contrasting incidence rate of scrub typhus, thereby suggesting a direction for preventive management of scrub typhus in Korea.

## Figures and Tables

**Table 1.** General characteristics of subjects

	Korea (n=406)	Japan (n=216)	p-value^1^
Ages (yr)			<0.001
50-59	93 (22.9)	49 (22.7)	
60-69	111 (27.3)	108 (50.0)	
70-79	202 (49.8)	59 (27.3)	
Sex			<0.001
Male	148 (36.5)	143 (66.2)	
Female	258 (63.5)	73 (33.8)	
Agricultural type			<0.001
Full-time	401 (98.8)	172 (79.6)	
Part-time	5 (1.2)	44 (20.4)	

Values are presented as number (%).

1 Data were analyzed using chi-square test.

**Table 2.** Characteristics of agricultural work and weeding

	Korea	Japan	p-value^1^
Agricultural work experience (yr)	43.2±17.4	30.5±17.5	
Agricultural work time (hr/d)	8.0±2.9	5.2±3.1	<0.001
Working posture during agriculture work^2^			
Stretching back with arm raised	1.6±1.0	1.4±0.8	0.18
Slight bending forward	2.3±1.1	2.1±0.9	0.07
Deep bending forward	3.0±1.0	2.1±1.0	<0.001
Kneeling or crouched position	3.0±1.1	2.1±1.0	<0.001
Rest place during agriculture work^3^			<0.001
Around farmland	274 (73.3)	65 (36.7)	
Home	88 (23.5)	63 (35.6)	
Agricultural machinery and car	6 (1.6)	44 (24.9)	
Etc.	6 (1.6)	5 (2.8)	
Proportion of weeding			
Agricultural	352 (86.7)	192 (88.9)	0.51
Residential	291 (71.7)	201 (93.1)	<0.001
Frequency of weeding (/2 mo)			
Agricultural	3.1±2.2	3.8±2.6	0.002
Residential	1.8±1.2	2.6±3.7	0.03

Values are presented as mean±standard deviation or number (%).

1 Data were analyzed using t-test and chi-square test.

2 4-point Likert scale.

3 Denominator: a person who rests during agricultural work.

**Table 3.** Knowledge related to scrub typhus

Items	Korea		Japan		p-value^1^
	Answer	Wrong answer	Answer	Wrong answer	
Percentage of overall correct answers	66.3±27.5		22.9±27.4		<0.001
Occurs in autumn^2^	264 (65.0)	142 (35.0)	20 (9.3)	196 (90.7)	<0.001
It is caused by mite bite	260 (64.0)	146 (36.0)	19 (8.8)	197 (91.2)	<0.001
One does not get infected in the farm house3	117 (28.8)	289 (71.2)	34 (15.8)	181 (84.2)	<0.001
Symptoms occur 1-2 weeks after being bitten by mites	198 (48.8)	208 (51.2)	26 (12.0)	190 (88.0)	<0.001
Symptoms are similar to colds (headache, high fever, chills)	295 (72.8)	110 (17.2)	33 (15.3)	183 (84.7)	<0.001
If you have symptoms, you should take a cold medicine and take a rest at home^3^	238 (58.8)	167 (41.2)	52 (24.2)	163 (75.8)	<0.001
Once infected, one does not get any more^3^	137 (33.7)	269 (76.3)	37 (17.1)	179 (82.9)	<0.001
If not cured, you could die	231 (56.9)	175 (43.1)	36 (16.7)	180 (83.3)	<0.001
Do not spread it to others^3^	56 (13.8)	350 (86.2)	4 (1.9)	212 (98.1)	<0.001
You should wear a hat and wrap a towel around your neck while farming	310 (76.4)	96 (23.6)	58 (26.9)	158 (73.1)	<0.001
You should wear long sleeve clothes, fabric wristlet and gloves while working on the farm	327 (81.3)	79 (18.7)	92 (41.4)	124 (58.6)	<0.001
You should wear long trousers and boots while working on the farm	328 (80.5)	78 (19.5)	80 (42.6)	135 (57.4)	<0.001
You should not put your clothes on weeds while working on the farm	330 (80.8)	76 (19.2)	82 (37.2)	134 (62.8)	<0.001
You should not sit or lie down on weeds while working on the farm	329 (81.0)	77 (19.0)	72 (38.0)	144 (62.0)	<0.001
After farming, you should take a shower or bath	327 (80.5)	79 (19.5)	75 (33.3)	141 (66.7)	<0.001
After farming, you should wash your clothes	334 (87.4)	44 (12.6)	88 (34.7)	137 (65.3)	<0.001
You do not need to wash clothes that have been treated with a mite repellent^3^	258 (63.7)	147 (46.3)	62 (28.7)	154 (71.3)	<0.001
Mite repellent can be sprayed once a day^3^	167 (41.1)	239 (58.9)	15 (6.9)	201 (93.1)	<0.001

Values are presented as mean±standard deviation or number (%).

1 Data were analyzed using t-test and chi-square test.

2 Considering the climate and harvesting time, there was a one-month gap between countries. This period is from September to November in Korea and from October to December in Japan.

3 Negative question.

**Table 4.** Behavior of agricultural work related to scrub typhus incidence^1^

Items	Korea	Japan	p-value^2^
Wear a hat during farming	4.5±1.0	4.8±0.5	<0.001
Wrap a towel around the neck during farming	2.9±1.7	3.6±1.1	<0.001
Wear long sleeve clothes and fabric wristlet during farming	4.6±0.8	4.8±0.5	0.001
Wear gloves during farming	4.6±0.9	4.6±0.6	0.88
Wear long trousers and socks during farming	4.9±0.4	4.9±0.4	0.55
Wear long boots during farming	4.0±1.3	4.5±0.8	<0.001
Put clothes on the weeds during farming^3^	4.3±1.1	4.5±0.7	0.002
Sit or lie down on weeds during farming^3^	3.8±1.2	4.2±0.8	<0.001
Wash clothes immediately after farming	4.3±0.9	4.6±0.6	<0.001
Take a shower or bath after farming	4.2±0.9	4.5±0.6	0.001
Total	4.2±0.5	4.5±0.3	<0.001

Values are presented as mean±standard deviation.

1 Data were measured on a 5-point Likert scale.

2 Data were analyzed using the t-test.

3 Negative question, calculated in reverse order.
