# Supplementary material for: Differences in agricultural activities related to incidence of scrub typhus between Korea and Japan
Source: Epidemiol Health. 2017 Nov 9;39:e2017051. doi: 10.4178/epih.e2017051 (PMC5790984; doi:10.4178/epih.e2017051)
Supplement: Supplementary file 1 [file epih-39-e2017051-supplementary.pdf]

Volume: 39, Article ID: e2017051

https://doi.org/10.4178/epih.e2017051

Differences in agricultural activities related to incidence of scrub typhus between Korea and Japan

Running title: Agricultural activities between Korea and Japan

Chang-Jin Ma[1], Gyung-Jae Oh[2], Gong-Unn Kang[3], Jeong Mi Lee[4], Da-Un Lee[5], Hae-Sung Nam[6], So Yeon Ryu[7], Young-Hoon Lee[2]

[1]Department of Environmental Science, Fukuoka Women's University, Fukuoka, Japan; [2]Department of Preventive Medicine and Public Health, Wonkwang University School of Medicine, Iksan, Korea; [3]Department of Medical Administration, Wonkwang Health Science University, Iksan, Korea; [4]Graduate School of Public Health, Wonkwang University, Iksan, Korea; [5]Department of Korean History Education, Wonkwang University College of Education, Iksan, Korea; [6]Department of Preventive Medicine and Public Health, Chungnam National University School of Medicine, Daejeon, Korea; [7]Department of Preventive Medicine, Chosun University Medical School, Gwangju, Korea

Correspondence: Gyung-Jae Oh

Department of Preventive Medicine and Public Health, Wonkwang University School of Medicine, 460 Iksandae-ro, Iksan, Jeonbuk 54538, Korea

E-mail: pmokj@wku.ac.kr

Volume: 39, Article ID: e2017051

https://doi.org/10.4178/epih.e2017051

Abstract

OBJECTIVES: The purpose of this study was to establish a basis for improving or strengthening the preventive strategy against scrub typhus in Korea by comparing and analyzing the difference of prevention behaviors contributing to the occurrence of scrub typhus in Japan and Korea.

METHODS: The survey was carried out in Jeollabuk-do, which is a high risk and high incidence area, and Fukuoka Prefecture, which is a high risk and low incidence area. The study included 406 Korean farmers and 216 Japanese farmers. Data were collected through face-to-face surveys by interviewers who had completed standardized education.

RESULTS: Korean farmers have a higher percentage of agricultural working posture that involved contact with weeds than Japanese farmers ($p<0.05$). The frequency and proportion of weeding were lower in Korean farmers than in Japanese farmers ($p<0.05$). The level of knowledge about scrub typhus was significantly higher among Korean farmers than among Japanese farmers ($p<0.05$). Mostly, the behavior of agriculture work was more appropriate for Japanese farmers than for Korean farmers ($p<0.05$). The total average level of agricultural work was lower in Korea than in Japan, lower in men than women, and lower in part-time farmers than full-time farmers ($p<0.05$).

CONCLUSIONS: This study suggests that it is reasonable to develop and provide a program that can improve the level of preventive behavior taking into consideration the characteristics of the subject in order to reduce the incidence of diseases in high-risk areas for scrub typhus.

KEY WORDS: Scrub typhus, Knowledge, Behavior, Agriculture, Korea, Japan

Volume: 39, Article ID: e2017051

https://doi.org/10.4178/epih.e2017051

INTRODUCTION

한국과 일본은 쯔쯔가무시증 발생 위험지역에 속해 있으며 특히 한국과 일본의 남부 지역은 기후, 생태학적 환경과 발생 환자들의 역학적 특성도 큰 차이가 없는 것으로 보고되고 있다[1-5]. 한국과 일본의 남부 지역의 쯔쯔가무시증 발생 환자들은 농작업 수행, 농업인, 여성, 60세 이상에서 많이 발생하였으며 시기적으로는 주로 가을에 발생하였다[3, 6]. 가을철은 농작물 수확과 관련된 농작업이 집중적으로 이뤄지는 시기적 특성과 발생 환자들이 공통적으로 농작업과 관련되어 있는 특성을 고려할 때 한국과 일본의 남부 지역의 쯔쯔가무시증 발생은 가을철 농작업 수행과 밀접하게 관련되어 있음을 알 수 있다.

한국과 일본의 남부 지역의 쯔쯔가무시증 발생의 역학적 특성들은 비슷하지만, 발생 현황에 있어서는 큰 차이를 나타내고 있다. 최근 10년('07-'16년도) 동안 누적 발생 건수와 발생률(10만명당)은 일본의 경우 각각 4,185명, 0.32로 낮은 수준이었으나 한국은 각각 75,658명, 14.92로 매우 높게 나타났다. 한국의 쯔쯔가무시증 발생 지표는 일본에 비해 발생 건수는 약 18배, 발생률은 약 47배 높은 수치로, 한국이 일본보다 쯔쯔가무시증으로 인한 질병부담은 더 컸다[7-9]. 한국에서 2000년 이후 쯔쯔가무시증이 지속적으로 증가하는 이유는 기후 온난화에 따른 병원체 및 매개체의 증가 및 인간의 노출 행태 증가와 관련이 있는 것으로 보고되고 있으며[10], 농업과 관련해서는 친환경 농작물 재배 증가, 밭 경작률 증가, 농업인구의 고령화 등을 고려해 볼 수 있다[11-14].

한편 쯔쯔가무시증은 털진드기 매개체에 의한 감염병이기 때문에 발생 예방과 관리는 매개체에 대한 접촉차단 및 접촉 회피를 통해 감염기회를 낮추는 방안을 우선적으로 고려해야 한다[15]. 한국과 일본의 남부 지역의 쯔쯔가무시증 발생에 영향을 미치는 환경이나 기후가 비슷하고 주요 매개체가 활순털진드기(*Leptotrombidium scutellare*)로 비슷한 상황을 고려할 때[1-6], 두 국가 간의 쯔쯔가무시증 발생률 차이는 사람과 매개체와의 접촉 관련 특성과 관련이 있을 것이다. 그러나 그 동안의 연구는 고위험군에서의 털진드기와의 접촉 차단을 통

Volume: 39, Article ID: e2017051

https://doi.org/10.4178/epih.e2017051

한 효과적인 쯔쯔가무시증 발생 예방 및 관리대책에 대한 근거를 뚜렷하게 제시하지 못하였고[5, 16], 적극적인 관리 정책을 진행하는 데에도 한계가 있었다.

따라서 한·일 두 국가의 쯔쯔가무시증 발생과 관련된 농작업 수행 행태나 환경 관리의 차이를 명확히 하고, 고위험군인 농업인과 털진드기와의 접촉을 차단할 수 있는 개입 방안을 마련할 수 있다면 한국에서의 쯔쯔가무시증 발생을 감소시킬 수 있을 것이다[17]. 이에 이 연구에서는 한·일 두 국가의 매개체와의 접촉차단 및 접촉 회피와 관련된 농작업 수행 및 행태에 대한 차이점을 명확히 하여 한국의 쯔쯔가무시증 발생을 억제할 수 있는 효과적인 예방관리의 지향점을 제시하고자 하였다.

MATERIALS AND METHODS

연구대상

대상 지역은 비교성을 높이기 위해 지리적 특성, 기후 및 농업 관련 특성 상 쯔쯔가무시증 발생 위험성이 높다는 공통점을 지니면서 발생 규모는 한국은 높고, 일본은 낮을 것으로 정하였다. 이러한 선정 기준에 따라 한국에서는 고위험, 고발생 지역인 전라북도와 일본은 고위험 저발생 지역인 후쿠오카현을 선정하였다.

전라북도와 후쿠오카현은 평야와 구릉 지형 분포가 유사한 것으로 나타났다. 후쿠오카현의 연평균 기온과 강수량 및 농경지 구성 비율은 규슈 지역 중 가장 전라북도와 비슷하였다[18-20]. 규슈 지역 전체는 지형 및 기후적 측면에서는 쯔쯔가무시증 위험 지역으로 분류되지만 후쿠오카현의 최근 10년간 쯔쯔가무시증 발생은 25건으로 매우 드물었으며 규슈 내 다른 지역들에 비해 가장 낮은 것으로 나타났다[7]. 이러한 특성을 고려할 때 후쿠오카현은 규슈 지역 중 지형 및 기후 특성은 전라북도와 가장 비슷하지만 쯔쯔가무시증 발생 현황은 서로 대조적이어서 쯔쯔가무시증 발생 관련 요인을 비교하기에 적절한 것으로 평가하였다[7,

Volume: 39, Article ID: e2017051

https://doi.org/10.4178/epih.e2017051

9].

조사 단위는 농업 특성과 농작업 환경, 지리적 환경 및 행태의 동질성을 유지하기 위해 마을로 정하였다. 마을은 보건기관 및 영농단체 전문가 토의 및 사전 현지 조사를 통해 지형, 농경지 및 재배 농작물, 조사 수행 가능성 및 대상자 참여 가능성 등을 파악한 후 판단 표본추출법(judgment sampling)을 이용하여 임의로 선정하였다. 조사 마을은 한국에서는 7개 마을(3 지역), 일본에서는 5개 마을(2 지역)이 선정되었다(Figure 1).

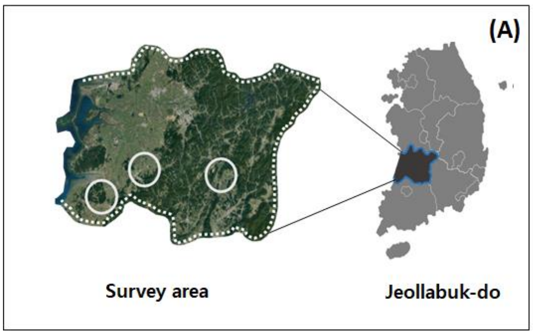

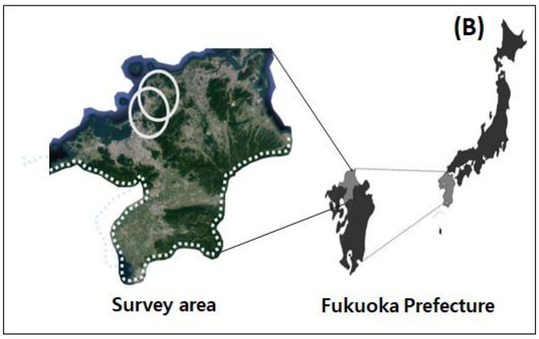

Figure 1. The surveyed areas consisted of 7 villages (3 regions-white open circles) in Jeollabuk - do in Korea (A) and 5 villages (2 regions-white open circles) in Fukuoka Prefecture in Japan (B). These areas were similar regarding agricultural characteristics, agricultural work environment, and topography.

대상자는 전체 마을 주민 중 농사 규모에 관계없이 현재 농업에 종사하고 있는 사람으로 한정하였다. 최종적으로 선정된 대상자는 한국 406명, 일본 216명이었다.

연구방법

자료 수집은 표준화 교육을 받은 조사원들에 의해 대면 조사를 통해 이뤄졌다. 조사 표

Volume: 39, Article ID: e2017051

https://doi.org/10.4178/epih.e2017051

준화를 위해 공통 설문도구와 조사 지침을 개발하여 활용하였으며 공통 교육 프로그램을 통해 조사원 교육을 수행하였다. 조사는 전라북도와 규슈 지역은 기후 및 쯔쯔가무시증이 발생이 10~11월에 집중되는 특성과 12월 전후로 농작물 수확이 종료되는 시기를 고려하여[3, 6, 21] 2014년 11월 말에서 12월 중순까지 1개월 동안 수행되었다.

설문의 준거 기간은 사전 조사를 통해 조사 시작일을 기점으로 하여 확정하였다. 농작업 특성 관련 내용은 농작물의 가을철 수확기로서 농작업 수행이 많은 시기라는 특성과 쯔쯔가무시증 발생 및 주요 매개체인 활순 털진드기(*L. scutellare*) 유충의 활동이 많은 시기인 9~11월을 고려하여 '최근 3개월'을 적용하였다. 제초 관련 내용은 제초 작업이 이뤄지는 시기를 사전에 확인하여 '최근 6개월'을 적용하였다. 설문도구 및 조사 지침은 표준 한글판을 개발한 후 일본어를 전공한 한국인을 통해 일본어로 일차 번역하였으며 번역된 일본어판은 한국어를 전공한 일본인을 통해 한국어로 재번역 하였다.

조사 내용은 인구사회학적 특성, 농작업 경력, 일평균 농작업 수행 시간, 쯔쯔가무시증에 대한 지식, 농작업 수행 행태, 제초 작업 특성 등으로 구성하였다. 쯔쯔가무시증에 대한 지식은 각 문항에 대한 정답률을 측정하였고 '모른다'고 대답한 경우는 오답으로 간주하였다. 쯔쯔가무시증 발생과 관련된 농작업 수행 수준은 Likert 5점 척도를 이용하여 구성하였다.

자료 분석

자료 분석은 SPSS version 21(IBM corp, Armonk, NY)을 이용하였다. 대상자의 일반적 특성, 농작업 중 휴식, 제초 비율, 지식 수준 등의 두 국가 간 분포 차이는 χ^2-test를 적용하여 파악하였으며, 농작업 경력과 농작업 자세와 제초 횟수, 지식 전체 점수, 농작업 행태 등의 두 국가 간 차이는 t-test를 적용하여 분석하였다. 대상자의 일반적 특성을 고려한 두 국가별 농작업 수행 행태 차이는 two-way ANOVA를 적용하여 파악하였다.

Volume: 39, Article ID: e2017051

https://doi.org/10.4178/epih.e2017051

Ethics statement

The present study protocol was reviewed and approved by the Wonkwang University Institutional Review Board (IRB no. WKIRB-201410-SB-059).

RESULTS

1. 대상자의 일반적 특성

대상자는 한국 406명, 일본 216명이었다. 한국은 70대가 202명(49.8%)으로 가장 많았고 일본은 60대가 108명(50.0%)로서 두 국가간 연령별 분포는 통계적으로 유의한 차이가 있었다(p<0.001). 성별에 따른 분포는 한국은 여자 63.5%, 일본은 남자 66.2%로서 통계적으로 유의한 차이가 있었다(p<0.001). 전업 농업인 비율 또한 한국은 98.8%, 일본은 79.6%로서 통계적으로 유의한 차이가 있었다(p<0.001)(Table 1).

Table 1. General characteristics of subjects

	Korea (n=406)	Japan (n=216)	p-value[1]
Ages (yr)			<0.001
50-59	93 (22.9)	49 (22.7)	
60-69	111 (27.3)	108 (50.0)	
70-79	202 (49.8)	59 (27.3)	
Sex			<0.001
Male	148 (36.5)	143 (66.2)	
Female	258 (63.5)	73 (33.8)	
Agricultural type			<0.001
Full-time	401 (98.8)	172 (79.6)	
Part-time	5 (1.2)	44 (20.4)	

Values are presented as number (%).

[1]Data were analyzed using chi-square test.

Volume: 39, Article ID: e2017051

https://doi.org/10.4178/epih.e2017051

2. 농작업 수행 및 제초 작업 특성

농작업 경력은 한국은 43.2년, 일본은 30.5년이었으며 하루 농작업 시간은 한국 8.0시간, 일본 5.2 시간으로 한국이 일본보다 더 긴 것으로 나타났다($p<0.001$). 농작업 수행 자세는 한국이 일본보다 허리를 많이 숙이거나 쪼그리기 및 무릎을 꿇은 자세 등 풀숲과의 접촉 가능한 자세 비율이 높았다($p<0.001$)(Table 2).

Table 2. Characteristics of agricultural work and weeding

	Korea	Japan	p-value[1]
Agricultural work experience (yr)	43.2 ±17.4	30.5 ±17.5	<0.001
Agricultural work time (hr/d)	8.0 ±2.9	5.2 ±3.1	<0.001
Working posture during agriculture work[2]			
Stretching back with arm raised	1.6 ±1.0	1.4 ±0.8	0.18
Slight bending forward	2.3 ±1.1	2.1 ±0.9	0.07
Deep bending forward	3.0 ±1.0	2.1 ±1.0	<0.001
Kneeling or crouched position	3.0 ±1.1	2.1 ±1.0	<0.001
Rest place during agriculture work[3]			<0.001
Around farmland	274 (73.3)	65 (36.7)	
Home	88 (23.5)	63 (35.6)	
Agricultural machinery and Car	6 (1.6)	44 (24.9)	
Etc.	6 (1.6)	5 (2.8)	
Proportion of weeding			
Agricultural	352 (86.7)	192 (88.9)	0.51
Residential	291 (71.7)	201 (93.1)	<0.001
Frequency of weeding (/2 mo)			
Agricultural	3.1 ±2.2	3.8 ±2.6	0.002
Residential	1.8 ±1.2	2.6 ±3.7	0.03

Values are presented as mean±standard deviation or number (%).

[1]Data were analyzed using t-test and chi-square test.

[2]4-point Likert scale.

[3]Denominator: a person who rests during agricultural work.

Volume: 39, Article ID: e2017051

https://doi.org/10.4178/epih.e2017051

농작업 중 휴식 장소는 한국은 농경지 주변 73.3%, 가정 23.5%의 분포를 보였으나 일본은 농경지 주변 36.7%, 가정 35.6%, 농기계나 자동차 안 24.9%의 분포로 국가간 차이를 나타내었다($p<0.001$)(Table 2).

제초 작업은 농경지의 경우 한국과 일본 모두 86-89%로 높아 차이가 없었으나 주거지 환경에서는 한국(71.7%)이 일본(93.1%)보다 낮게 나타났다($p<0.001$). 제초 횟수(/2개월)는 농경지의 경우 한국(3.1회)이 일본(3.8회)보다 낮았으며 주거지 환경에서도 한국(1.8회)이 일본(2.6회)보다 낮게 나타났다($p<0.05$)(Table 2).

3. 쯔쯔가무시증에 대한 지식 수준

쯔쯔가무시증에 대한 전반적인 지식(발생 시기, 전파경로, 위험요인, 임상 증상, 사후관리 및 기타 역학적 특성, 주요 예방관련 행태, 진드기 기피제 사용 등) 수준은 일본에 비해 한국에서 상대적으로 더 높은 것으로 나타났다($p<0.001$). 한국은 발생 시기, 전파 매개체, 증상, 예후, 안전한 농작업 착용, 사후 관리 등 대부분의 항목에서 60-90점으로 높은 정답률을 나타내었다, 그러나 집안에서의 농작업, 잠복기간, 재발가능성, 사람 간 전파, 진드기 기피제 사용방볍 등의 항목에서는 60점미만으로 낮게 나타났다. 일본은 대부분의 항목에서 30점미만으로 낮았으나 안전한 농작업복 착용 및 사후관리 등에서는 30-50점으로 다른 항목에 비해 높은 것으로 나타났다. 특히, 안전한 농작업복 착용과 농작업복 세탁 및 목욕 등 개인위생과 관련된 지식에서는 한국과 일본 모두 각각의 국가 전체 평균에 비해 높게 나타났다(Table 3).

Volume: 39, Article ID: e2017051

https://doi.org/10.4178/epih.e2017051

Table 3. Knowledge related to scrub typhus

Items	Korea		Japan		p-value[1]
	Answer	Wrong answer	Answer	Wrong answer	
Percentage of overall correct answers	66.3 ±27.5		22.9 ±27.4		<0.001
Occurs in autumn[2]	264 (65.0)	142 (35.0)	20 (9.3)	196 (90.7)	<0.001
It is caused by mite bite	260 (64.0)	146 (36.0)	19 (8.8)	197 (91.2)	<0.001
One does not get infected in the farm house[3]	117 (28.8)	289 (71.2)	34 (15.8)	181 (84.2)	<0.001
Symptoms occur 1-2 weeks after being bitten by mites	198 (48.8)	208 (51.2)	26 (12.0)	190 (88.0)	<0.001
Symptoms are similar to colds (headache, high fever, chills)	295 (72.8)	110 (17.2)	33 (15.3)	183 (84.7)	<0.001
If you have symptoms, you should take a cold medicine and take a rest at home[2]	238 (58.8)	167 (41.2)	52 (24.2)	163 (75.8)	<0.001
Once infected, one does not get any more[3]	137 (33.7)	269 (76.3)	37 (17.1)	179 (82.9)	<0.001
If not cured, you could die	231 (56.9)	175 (43.1)	36 (16.7)	180 (83.3)	<0.001
Do not spread it to others[3]	56 (13.8)	350 (86.2)	4 (1.9)	212 (98.1)	<0.001
You should wear a hat and wrap a towel around your neck while farming	310 (76.4)	96 (23.6)	58 (26.9)	158 (73.1)	<0.001
You should wear long sleeve clothes, fabric wristlet and gloves while working on the farm	327 (81.3)	79 (18.7)	92 (41.4)	124 (58.6)	<0.001
You should wear long trousers and boots while working on the farm	328 (80.5)	78 (19.5)	80 (42.6)	135 (57.4)	<0.001
You should not put your clothes on weeds while working on the farm	330 (80.8)	76 (19.2)	82 (37.2)	134 (62.8)	<0.001
You should not sit or lie down on weeds while working on the farm	329 (81.0)	77 (19.0)	72 (38.0)	144 (62.0)	<0.001
After farming, you should take a shower or bath	327 (80.5)	79 (19.5)	75 (33.3)	141 (66.7)	<0.001
After farming, you should wash your clothes	334 (87.4)	44 (12.6)	88 (34.7)	137 (65.3)	<0.001
You do not need to wash clothes that have been treated with a mite repellent[2]	258 (63.7)	147 (46.3)	62 (28.7)	154 (71.3)	<0.001
Mite repellent can be sprayed once a day[3]	167 (41.1)	239 (58.9)	15 (6.9)	201 (93.1)	<0.001

Values are presented as mean±standard deviation or number (%);

[1]Data were analyzed using t-test and chi-square test.

[2]Considering the climate and harvesting time, there was a one-month gap between countries. This period is from September to November in Korea and from October to December in Japan.

[3]Negative question

Volume: 39, Article ID: e2017051

https://doi.org/10.4178/epih.e2017051

4. 농작업 수행 행태

전반적인 농작업 수행 수준은 한국이 일본보다 낮게 나타났다(p<0.001). 특히 모자 착용, 목에 수건 두르기, 긴팔 옷 및 토시 착용, 장화신기, 풀위에 옷 벗어 놓지 않기, 풀밭에 앉거나 눕지 않기, 농작업 후 농작업복 세탁, 농작업 후 샤워 또는 목욕하기 등, 대부분의 항목에서 한국이 일본보다 수행 수준이 낮게 나타났다(P<0.001). 목에 수건 두르기는 두 국가 모두 다른 항목에 비해 수행 수준이 낮게 나타났다. 한편 농작업 시 장갑 착용, 긴바지 및 양말 착용 항목은 두 국가 간 차이가 없는 것으로 나타났다(Table 4).

Table 4. Behavior of agricultural work related to scrub typhus incidence[1]

Items	Korea	Japan	p-value[2]
Wear a hat during farming	4.5 ±1.0	4.8 ±0.5	<0.001
Wrap a towel around the neck during farming	2.9 ±1.7	3.6 ±1.1	<0.001
Wear long sleeve clothes and fabric wristlet during farming	4.6 ±0.8	4.8 ±0.5	0.001
Wear gloves during farming	4.6 ±0.9	4.6 ±0.6	0.88
Wear long trousers and socks during farming	4.9 ±0.4	4.9 ±0.4	0.55
Wear long boots during farming	4.0 ±1.3	4.5 ±0.8	<0.001
Put clothes on the weeds during farming[3]	4.3 ±1.1	4.5 ±0.7	0.002
Sit or lie down on weeds during farming[3]	3.8 ±1.2	4.2 ±0.8	<0.001
Wash clothes immediately after farming	4.3 ±0.9	4.6 ±0.6	<0.001
Take a shower or bath after farming	4.2 ±0.9	4.5 ±0.6	0.001
Total	4.2 ±0.5	4.5 ±0.3	<0.001

Values are presented as mean±standard deviation.

1Data were measured on a 5-point Likert scale.

2Data were analyzed using the t-test

3Negative question, calculated in reverse order.

Volume: 39, Article ID: e2017051

https://doi.org/10.4178/epih.e2017051

전반적인 농작업 수행 행태는 모든 연령대에서 한국이 일본보다 낮았다(P<0.001). 한국에서는 60대에서 가장 높고, 50대와 70대에서는 낮게 나타났으나 일본에서는 연령이 증가할수록 높게 나타났다(p<0.001). 성별에 따른 전반적인 농작업 수행 행태는 남성과 여성 모두 한국이 일본보다 낮았으며 두 국가 모두 여성이 남성보다 높게 나타났다(p<0.001). 한편 농업 전담 여부에 관계없이 전반적인 농작업 수행 행태는 한국이 일본보다 낮았으며 한국에서는 겸업 농업인에서 전업 농업인보다 낮은 것으로 나타났다(p<0.05). 그러나 일본에서는 겸업 농업인과 전업 농업인의 농작업 수행 행태는 차이가 없는 것으로 나타났다(Figure 2).

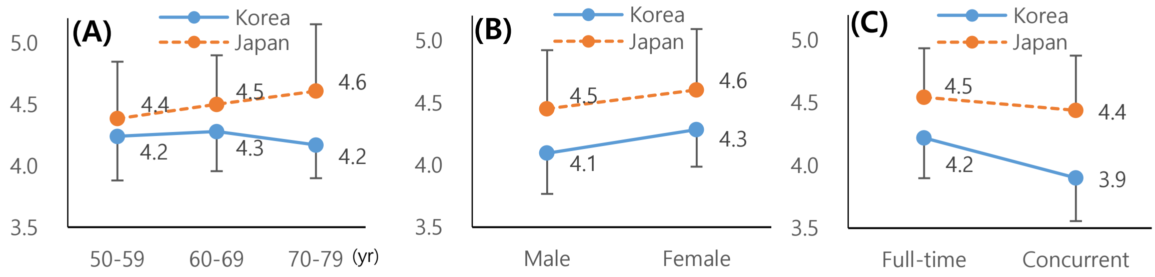

Figure 2. Differences in total average level of behavior of agriculture work according to general characteristics and country. Data were expressed as the means and standard deviation. Statistical analysis were performed using two-way ANOVA to determine the main effects and interaction between variables. **(A)** $F_{country}$ = 47.044, $p < 0.001$; F_{ages} = 1.503, p = 0.22; $F_{country \times ages}$ = 4.978, p = 0.007. **(B)** $F_{country}$ = 77.280, $p < 0.001$; F_{gender} = 19.568, $p <$ 0.001; $F_{country \times gender}$ = 0.224, p = 0.64. **(C)** $F_{country}$ = 18.541, $p < 0.001$; $F_{agricultural\ type}$ = 4.659, $p < 0.05$; $F_{country \times agricultural\ type}$ = 2.953, p = 0.09.

DISCUSSION

일반적으로 농업인들은 고연령 일수록 면역능력이 낮아 감염에 대한 저항력이 낮고 체력이 약해 농작업 현장 근처에서 자주 휴식하게 된다. 또한 여성 농업인은 털진드기가 서식하는 풀숲 환경에서 수작업으로 이뤄지는 밭농사를 주로 담당하기 때문에 매개체인 털진드기와 접촉할 확률이 높게 된다[13, 22]. 밭농사는 대부분 쪼그려 앉거나 허리를 구부리는 작업

자세를 취해야 하며 이러한 작업 자세는 사람이 풀숲, 작물, 토양에 서식하는 털진드기에 쉽게 접촉하게 하고 물릴 가능성을 증가시킨다[22].

대상자들은 한국에서 일본보다 고령화 되어 있고, 여성 및 전업 농업인 비율이 높으며 농작업 수행 시간은 긴 것으로 나타났다. 또한 한국에서의 농작업 수행 자세는 일본보다 허리를 숙이거나 쪼그려 앉은 자세 비중이 더 높은 것으로 나타났다. 따라서 한국의 농업인들은 인구학적 특성 및 농작업 수행 특성 상 일본에 비해 털진드기와의 접촉 가능성이 높고, 쯔쯔가무시증 발생 위험 요인이 더 높을 수 있음을 알 수 있다.

농경지나 농경지 주변 그리고 농촌의 주거지 주변은 농업인의 생활공간이지만 농작물이나 풀숲이 우거져 털진드기 서식에 적합하여 사람과 털진드기의 접촉이 이뤄지는 주요 장소이다[22, 23]. 한국에서의 농작업 중 휴식은 대부분 논・밭 두렁 등 농경지 주변에서 이뤄지고 있어 자동차 안이나 주거지에서 휴식하는 일본 농업인과는 다른 양상으로 나타나고 있었다. 논・밭두렁은 쯔쯔가무시증을 매개하는 털진드기가 서식하는 주요 장소로 이곳에서 농작업 중 휴식하는 것은 사람과 털진드기와의 접촉 기회를 증가시키게 된다[24].

털진드기는 쯔쯔가무시균 감염률이 1-2%로 매우 낮고, 특정 지점에 군집하여 서식하는 특성(mite island)을 갖고 있다[16, 25, 26]. 이러한 특성 때문에 쯔쯔가무시균에 감염된 털진드기를 특정해서 파악하기 어렵고 정확하게 털진드기 서식지를 파악하는 것도 어렵다. 따라서 쯔쯔가무시증 예방을 위해 털진드기를 방제하는 것은 실효성이 낮을 수 밖에 없다. 또한 털진드기 방제 물질 구입에 따른 경제적 부담, 방제 후 초래될 수 있는 환경오염을 고려한다면 현장에서 방제 적용은 신중하게 접근해야 할 것이다.

이러한 이유로 쯔쯔가무시증을 예방하기 위해서는 털진드기 방제보다는 생활공간에서의 털진드기 서식지를 제거해 사람과 털진드기의 접촉 기회를 낮추는 것이 효율적일 수 있다[27]. 연구 결과에서도 쯔쯔가무시증 발생률이 높은 한국은 발생률이 낮은 일본보다 주거지 환경의 제초작업 수행이 미흡한 것으로 나타나고 있어 주거지 주변의 제초작업을 통한 털진

드기 서식지 제거는 쯔쯔가무시증 예방을 위한 하나의 방안으로 고려할 수 있겠다.

진드기 매개 감염병은 매개체인 진드기와의 접촉을 피하고 체내 침투한 진드기를 제거하는 등의 예방수칙을 준수하는 것이 가장 효과적인 예방 방법으로 알려져 있다[15, 28]. 이 연구에서 농작업 수행 수준은 전반적으로 한국이 일본보다 낮게 나타났다. 특히 피부 노출을 최소화한 농작업복 착용, 풀숲과 접촉을 피함으로써 털진드기와 접촉을 차단할 수 있는 농작업 수행 자세, 농작업 후 농작업복 세탁이나 몸씻기 등의 개인위생을 통한 의복이나 피부에 붙어 있는 털진드기 제거 수준에서 한국에서 상대적으로 더 낮게 나타났다. 따라서 한국 농업인들은 털진드기와의 접촉이나 털진드기의 체내 침투 기회가 많기 때문에 털진드기에 물릴 위험성이 높을 수 있다. 이렇듯 쯔쯔가무시증 발생률이 높은 한국에서 농작업 수행 수준이 상대적으로 낮은 결과는 한국에서 쯔쯔가무시증 발생률이 높은 이유를 농작업 수행 행태를 통해 설명할 수 있음을 보여주고 있다.

이 연구 대상 지역인 일본의 후쿠오카 현은 한국의 전라북도보다 연평균 기온(2012년도)이 약 4 ℃ 높음에도 불구하고[20] 농작업복 착용 시 피부 노출은 한국보다 일본에서 더 낮게 나타나고 있다. 또한 털진드기와 접촉 가능성이 높은 70대, 여성, 전업 농업인의 농작업 수행 수준은 발생률이 높은 한국에서 발생률이 낮은 일본보다 낮게 나타났다. 이러한 연구 결과는 농작업 수행 수준은 쯔쯔가무시증 위험 요인 및 발생 수준과 서로 다른 양상으로 나타날 수 있음을 제시해 주고 있다. 따라서 농작업 수행 행태들은 쯔쯔가무시증에 대한 인식이나 기후적 요인 외에도 두 국가의 사회·문화적 특성이 일정 부분 영향을 미쳐 형성될 것으로 이해할 수 있다. 건강과 관련된 특정 행태는 오랜 기간 동안 사회·문화적 특성의 영향을 받아 습관으로 고착된다고 알려져 있다[29]. 쯔쯔가무시증 예방을 위한 농작업 행태 개선은 고위험 지역 및 고위험군이 갖고 있는 사회·문화적 특성을 적극적으로 반영하여야 적정 수준의 예방 효과를 기대할 수 있을 것이다.

농작업 수행 수준이 일본에서는 연령 증가에 따라 높아지고 있으나 한국에서는 60 대에

Volume: 39, Article ID: e2017051

https://doi.org/10.4178/epih.e2017051

비해서 50대와 70대에서 낮게 나타나고 있었다. 따라서 한국에서는 상대적으로 젊거나 고령층에 대한 관리에 더 집중할 필요가 있으며 이들에서 적절한 농작업 수행을 어렵게 하는 장애요인을 파악하고 해결방안을 강구할 필요가 있다.

한·일 두 국가 모두에서 남성 농업인이나 겸업 농업인의 농작업 수행 수준은 여성 농업인 및 전업 농업인보다 낮게 나타났다. 한국과 같은 고위험 지역 농업인은 누구나 쯔쯔가무시증 위험 요인에 노출될 가능성이 높다. 따라서 고위험 지역 남성이나 겸업 농업인의 낮은 농작업 수행 수준은 쯔쯔가무시증 발생률을 증가시킬 가능성이 있으므로 쯔쯔가무시증 발생 고위험 지역에서의 발생 예방을 위한 개입 대상에는 이들을 포함해야 할 것이다.

한편 연구 결과에서 한국은 일본에 비해 쯔쯔가무시증에 대한 지식 수준은 높으나 농작업 수행 수준은 상대적으로 낮은 것으로 나타나고 있었다. 한국은 일본에 비해 쯔쯔가무시증 발생 수준이 높게 유지되고 있어 한국의 고발생 지역 주민의 경우 질환 인지도가 높고 질환에 대한 교육 및 홍보 경험이 상대적으로 많아서 지식 수준이 높게 나타난 것으로 이해된다. 이러한 결과는 한국은 쯔쯔가무시증에 대한 높은 지식 수준이 쯔쯔가무시증 발생 예방활동 수행으로 적절하게 연계되지 못하고 있음을 보여 주고 있다. 진드기 매개질환에 대한 지식수준이 높은 경우, 교육은 예방수칙 준수율을 향상시키는 데 가장 효과적인 개입방법으로 보고되고 있다[17, 30, 31]. 따라서 한국과 같이 쯔쯔가무시증 고위험 지역에서 발생 수준을 감소시키기 위해서는 쯔쯔가무시증에 대한 지식뿐만 아니라 예방수칙 준수율을 향상시킬 수 있는 내용을 핵심으로 하는 교육 프로그램의 개발과 제공이 필요함을 알 수 있다.

이 연구는 쯔쯔가무시증 발생에 영향을 미치는 요인 중 사람의 행태적 특성에 집중하였기 때문에 매개체 및 최종 숙주의 분포와 서식, 기후 등을 고려한 예방 대책에 대해서는 제시하지 않았다. 이 연구는 확률표본추출법을 적용하지 못하고 판단표본추출법을 이용하여 조사목적에 적합한 지역과 대상자를 임의적으로 선정하였기 때문에 표본의 대표성이 높지 않다는 한계를 지니고 있다. 또한 농작업 수행 행태가 한국에서 일본보다 낮은 결과는 대상

Volume: 39, Article ID: e2017051

https://doi.org/10.4178/epih.e2017051

자의 인구학적 특성이 두 국가에서 다르기 때문에 연령, 성별 및 농작업 참여 정도가 영향을 미쳤을 수 있음을 고려하여야 한다.

그럼에도 이 연구는 쯔쯔가무시증 발생 위험은 공통적이나 발생 현황에서는 차이가 있는 한·일 두 국가 농업인의 환경 관리 및 털진드기 접촉과 관련된 행태적 차이를 파악함으로써 한국의 쯔쯔가무시증 발생을 억제할 수 있는 예방관리의 방향을 제시하였다는 데 그 의의가 있다.

ACKNOWLEDGMENTS

This study was supported in 2016 by a research grant from the Wonkwang University**.**

Volume: 39, Article ID: e2017051

https://doi.org/10.4178/epih.e2017051

REFERENCES

1. World Health Organization. Frequently asked questions: scrub typhus [cited 2017 Sep 11]. Available from: http://www.searo.who.int/entity/emerging_diseases/CDS_faq_Scrub_Typhus.pdf.

2. Kang GU, Ma CJ, Oh GJ. Association between scrub typhus outbreaks and meteorological factors in Jeollabuk-do province. Korean J Environ Health Sci 2016;42:41-52 (Korean).

3. Korea Centers for Disease Control and Prevention. Epidemiological characteristics of patients with tsutsugamushi disease in 2013. Cheongju: Korea Centers for Disease Control and Prevention; 2014, p. 2-10 (Korean).

4. Ogawa M, Hagiwara T, Kishimoto T, Shiga S, Yoshida Y, Furuya Y, et al. Scrub typhus in Japan: epidemiology and clinical features of cases reported in 1998. Am J Trop Med Hyg 2002;67:162-165.

5. Korea Centers for Disease Control and Prevention. Study of risk factors for scrub typhus infection in highly endemic rural and urban area. Cheongju: Korea Centers for Disease Control and Prevention; 2013, p. 96-97 (Korean).

6. Oita Hygiene Environment Research Center. Tsutsugamushi disease in Oita prefecture, 1998-2007 [cited 2017 Sep 5]. Available from: http://www.pref.oita.jp/uploaded/attachment/12018.pdf (Japanese).

7. National Institute of Infectious Diseases. Infectious agents surveillance report [cited 2017 Sep 5]. Available from: https://www.niid.go.jp/niid/en/basic-science/865-iasr.html.

8. Korea Centers for Disease Control and Prevention. Infectious diseases surveillance

yearbook, 2015. Cheongjug: Korea Centers for Disease Control and Prevention; 2016, p. 32-33 (Korean).

9. Korea Centers for Disease Control and Prevention. Disease web statistics system [cited 2017 Sep 12]. Available from: http://is.cdc.go.kr/dstat (Korean).

10. Kim SJ. Epidemiological characteristics and environmental factors associated with scrub typhus outbreaks in Korea [dissertation]. Suwon: Ajou University; 2015 (Korean).

11. Kim S, Kim JS, Lee H. Epidemiological characteristics of scrub typhus in Korea, 2009. Osong Public Health Res Perspect 2010;1:55-60.

12. Kim DM, Kim KY, Nam HS, Kweon SS, Park MY, Ryu SY. Risk factors for human infection with Orientia tsutsugamushi: a case control study in Korea. Clin Microbiol Infect 2008;14:174-177.

13. Kong WS, Shin E, Lee HI, Hwang TS, Kim HH, Lee NY, et al. Time-spatial distribution of scrub typhus and its environmental ecology. J Korean Geogr Soc 2007;42:82-95 (Korean).

14. Ryu SY. Epidemiologic study for tsutsugamushi disease in Korea. Seoul: Korea Centers for Disease Control and Prevention; 2006, p. 29-31 (Korean).

15. Shadick NA, Daltroy LH, Phillips CB, Liang US, Liang MH. Determinants of tick-avoidance behaviors in an endemic area for Lyme disease. Am J Prev Med 1997;13:265-270.

16. Korea Centers for Disease Control and Prevention. Serosurveillance of scrub typhus on residents and Orientia tsutsugamushi infection rate on mite in high prevalence region. Cheongju: Korea Centers for Disease Control and Prevention; 2013, p. 53-54 (Korean).

17. Daltroy LH, Phillips C, Lew R, Wright E, Shadick NA, Liang MH. A controlled trial of a novel primary prevention program for Lyme disease and other tick-borne illnesses. Health Educ Behav 2007; 34:531-542.

18. Jeollabuk-do. General status of Jeollabuk-do [cited 2017 Sep 13]. Available from: http://www.jeonbuk.go.kr/index.jeonbuk?menuCd=DOM_000000105003002000 (Korean).

19. Jeonbuk Research Institute. A study on the analysis of agricultural structure in Jeollabuk-do. Jeonju: Jeonbuk Research Institute; 2014, p. 215-216 (Korean).

20. Fukuoka Prefecture. Fukuoka data web [cited 2017 Sep 13]. Available from: http://www.pref.fukuoka.lg.jp/dataweb/search/1/1007_388.html (Japanese).

21. Farm Station Fukuoka. Fukuoka prefecture agriculture, forestry and fisheries branding promotion council [cited 2017 Sep 13]. Available from: http://www.fs-fukuoka.com/catalog/catalog_vegetables (Japanese).

22. Korea Centers for Disease Control and Prevention. Epidemiology and management of tsutsugamushi disease. Cheongju: Korea Centers for Disease Control and Prevention; 2016, p. 14-15 (Korean).

23. Korea Centers for Disease Control and Prevention. Study of endemic foci of chigger mite as a vector of tsutsugamushi disease. Seoul: Korea Centers for Disease Control and Prevention; 2011, p. 77-80 (Korean).

24. Song HJ. Environmental survey on the vectors and hosts of tsutsugamushi disease in Jeonnam province, Korea. Korean J Vet Serv 2012;35:183-189 (Korean).

25. Korea Centers for Disease Control and Prevention. Determination of vector species

of tsutsugamushi disease and their geographical distribution in Korea. Public Health Wkly Rep 2009;2:1-5 (Korean).

26. Korea Centers for Disease Control and Prevention. Characteristics and geographical distribution of chigger mites as the vector of Orientia tsutsugamushi in Korea. Public Health Wkly Rep 2012;5:234-238 (Korean).

27. Hengbin G, Min C, Kaihua T, Jiaqi T. The foci of scrub typhus and strategies of prevention in the spring in Pingtan Island, Fujian Province. Ann N Y Acad Sci 2006;1078:188-196.

28. European Centre for Disease Prevention and Control. Personal protective measures against tick bites [cited 2017 Sep 11]. Available from: https://ecdc.europa.eu/en/disease-vectors/preventionand-control/protective-measures-ticks.

29. Global Health Education Consortium. Social & cultural factors related to health [cited 2017 Sep 11]. Available from: https://www.cugh.org/sites/default/files/13_Social_And_Cultural_Factors_Related_To_Health_Part_A_Recognizing_The_Impact_-_Copy_0.pdf.

30. Malouin R, Winch P, Leontsini E, Glass G, Simon D, Hayes EB, et al. Longitudinal evaluation of an educational intervention for preventing tick bites in an area with endemic lyme disease in Baltimore County, Maryland. Am J Epidemiol 2003;157:1039-1051.

31. Herrington JE Jr, Campbell GL, Bailey RE, Cartter ML, Adams M, Frazier EL, et al. Predisposing factors for individuals’ Lyme disease prevention practices: Connecticut,

https://doi.org/10.4178/epih.e2017051

Maine, and Montana. Am J Public Health 1997;87:2035-2038.
